# Milk and Milk-Contracts
*Previous articles appeared on May 3, 10, 17, 24, and 31.


**Published:** 1913-06-14

**Authors:** Everitt E. Norton

**Affiliations:** Medical Superintendent of Isleworth Infirmary.


### MILK AND MILK-CONTRACTS.*

By EVERITT E. NORTON, M.D., D.P.H., etc., Medical Superintendent of Isleworth Infirmary.

### VI.—The Comparative Values of Heated and Raw Milk.

It is, of course, not to be denied that milk may contain, amongst other varieties, pathogenic organisms either derived from the cow or introduced by human agency. The occurrence of epidemics of disease proved to have been milk-borne, the recognition of inflammatory and other diseases of the udder in milch-cows, and discussions as to the causation of tubercular disease in infants have all had a part in arousing suspicion as to milk and in popularising that custom of heating which tends to restore confidence in it. With regard to tubercle it is useless, in view of the conclusions of the Royal Commission and of other opinions, to deny that there is danger of infection by milk, and, in spite of optimistic paragraphs which from time to time appear in newspapers, it is a fact that routine examinations of milk carried out in London for the London County Council prove that it is actually infected by tubercle in no less than 10.8 per cent. of the samples examined (*The Lancet*, April 19, 1913).

On the other hand, there is also clear evidence that the heating of milk is injurious to it as a food, and that the damage done is roughly proportionate to the degree of temperature attained and the length of time of exposure to that temperature. Milk allowed to become stale or polluted and subsequently heated to render it " fit for use," or fit for sale, is doubly damaged; whilst organisms, or even spores, may be destroyed, *toxins remain*. Milk twice heated, first by the dairyman and afterwards by the purchaser, is necessarily so much the more reduced in food value.

### The Ill-Effects of Pasteurisation.

The demand for pasteurised milk which has arisen on the part of the public in America results not from a preference for such milk but from confidence in its safety. Some American authorities " advocate compulsory pasteurisation, not because they think pasteurised milk is better than clean raw milk, but because they consider that the bulk of raw milk, as at present produced, is liable to contain pathogenic bacteria " (Eastwood). Whilst for adult consumption the ill-effects of pasteurising or sterilising milk may possibly be not great, they are of the greatest moment in the matter of infant-feeding. No more definite adverse criticism of London milk could be propounded than the consensus of opinion that it is unsuitable and unsafe, without an attempt by means of heat to render it innocuous for the feeding of infants. It is common in institutions for a separate special supply of sterilised milk to be obtained for infants.

To enter here into the discussion concerning the relative values in infant-feeding of raw milk and of milk treated by heat would be to go beyond the limits of my present subject. Suffice it to say that general experience proves only too plainly that of the many modifications of cooked or partially cooked cow's milk in use not one is universally suitable or satisfactory; hence the widespread reliance which is placed in tinned condensed milks and in the multifarious milk-substitutes so freely advertised.

Much may be said, no doubt justifiably, in favour of the use as the basis of infants' food of the raw milk of the cow. Many of those who advise the use for infants of milk sterilised, or sterilised and modified, admit the disadvantages of the processes involved. It is an interesting fact that some of those engaged in the sale of sterilised prepared milks find it necessary to advocate the use with them of " antiscorbutic " substances, and I cannot refrain from quoting (from *The Lancet* of September 16, 1911) the remark of Dr. Eric Pritchard: " I have never seen an infant uninterruptedly fed for six months on condensed milk, which is, of course, milk which has been vigorously sterilised, who did not present unmistakable symptoms of rickets." We have yet much to learn concerning the whey-proteins of milk and the changes, affecting infant digestion, which occur when they are modified by heat: we know that in cow's milk the proportions of lactalbumin and extractives are low in comparison with human milk, and that lactalbumin (the chief constituent of the whey-proteins) coagulates at 161.6° F. The value of lactalbumin has of late been much insisted upon, and there has been a ready sale for " Albulactin," which is widely used as an infant-food adjuvant.

### Need for a Reliable Raw Milk Supply.

My object is to emphasise the need for a reliable supply of raw milk and the right of the consumer to know whether the milk sold to him has or has not been pasteurised. In the City of New York, amongst other rules added (in 1908) to the Sanitary Code, are instructions that pasteurised milk must bear the date and hour of pasteurisation and must be delivered to the consumer within twenty-four hours, and, further, that no milk shall be pasteurised a second time.

Dr. Newsholme, referring to the fact that dairymen now largely supply pasteurised milk, remarks that pasteurisation is " often done to preserve stale milk, and the slight taste of pasteurised milk is concealed by mixing the milk with fresh unpasteurised milk. This obviously gives little protection to the purchaser. Furthermore, the dairyman is only concerned in pasteurising at the lowest temperature which will prevent souring of the milk, a temperature which does not suffice to kill the tubercle bacillus. If, therefore, commercial pas-

* Previous articles appeared on May 3, 10, 17, 24, and 31.

teurised milk is to be regarded as safe in respect of tuberculosis, the temperature and duration of the heating process must be specified." Similarly in the case of institution milk supplies a final objection to the process of pasteurisation as at present carried out is its uncertainty. No evidence whatever is brought forward to prove that it is practically efficient in destroying tubercle or other infections, and it is doubtful in the extreme whether by many of the slipshod methods in vogue any real degree of safety is attained, or whether the employment of such methods is of any practical benefit whatever to the consumer.

### Revision of Milk-Contracts.

The facts may, I think, be acknowledged that many existing milk-contracts need amendment, and that the milk-trade has not yet realised the advantages which would accrue to itself from ameliorated methods.

Whilst public health legislation has undoubtedly effected something, the provisions have been largely permissive and have been hampered by limitations. In 1907 the London County Council obtained powers which may be summarised thus:—

(*a*) To sample milk sent to London railway stations or elsewhere.

(*b*) To submit milk to examination for the presence of tuberculous infection.

(*c*) To cause veterinary inspection to be made of the cows at the farm from which any such tuberculous milk is derived.

(*d*) To cause cows, which the veterinary inspector has reason to believe to have tuberculous udders, to be isolated and to prevent their milk being sent to London.

Elsewhere than in London some progressive steps are being taken. In Birmingham an attempt has been initiated to eliminate tuberculosis from particular herds supplying the city by "the Bang method," and the results show that so long as customers are willing to pay a slight increase in price over that of ordinary milk (how instructive is the use of the word "ordinary"!) a tubercle-free supply is obtainable; "it would now appear to rest with the public whether the movement is to progress or not." The excellent example which has been set by the American Milk Commissions has not yet been imitated in England. A Vice-regal Commission has been holding an investigation concerning the production and distribution of Irish milk supplies: it is reported that Professor McWeeney, giving evidence before that Commission, gave as his opinion that no person should be allowed to handle milk in a dairy or to milk cows without having first passed a Widal test, and that every clinically tuberculous cow discovered should be destroyed; excellent ideals, the former of which, at least, appears to be at present unattainable.

### Mr. John Burns' Pure Milk Bill.

There is now before the House of Commons a "Milk and Dairies Bill," commonly known as Mr. John Burns' Pure Milk Bill. Certain provisions of this Bill supersede some of those contained in the Contagious Diseases (Animals) Acts and the Orders made under them, and reproduce and amend sections of the Infectious Diseases (Prevention) Act, 1890, the Public Health (London) Act, 1891, and the milk clauses incorporated in many local Acts. The Bill deals with registration, inspection, the sale of tuberculous or other infected milk, imported milk, and "the issue of regulations for securing the supply of pure and wholesome milk." It empowers the Board of Agriculture and Fisheries to pay compensation for animals slaughtered in accordance with any order or regulation dealing with animal tuberculosis.

Education of the public is slowly proceeding. Newspaper articles appear (spasmodically) from time to time; the *Referee* of March 3, 1912, contained a vehement protest by Mr. Geo. R. Sims concerning the custom of pasteurising milk. Excellent leaflets, by the Joint Committee on Milk of the National Health Society and the National League for Physical Education and Improvement, have been published by the League from 4 Tavistock Square, W.C.

